# Muscle Activation and Kinematic Analysis during the Inclined Leg Press Exercise in Young Females

**DOI:** 10.3390/ijerph17228698

**Published:** 2020-11-23

**Authors:** Isabel Martín-Fuentes, José M. Oliva-Lozano, José M. Muyor

**Affiliations:** 1 Health Research Centre, University of Almería, 04120 Almería, Spain; imf902@ual.es (I.M.-F.); jol908@ual.es (J.M.O.-L.); 2 Laboratory of Kinesiology, Biomechanics and Ergonomics (KIBIOMER Lab.), Research Central Services, University of Almería, 04120 Almería, Spain

**Keywords:** knee muscle imbalances, muscle activation, surface electromyography, leg press variants, lower limb kinematics

## Abstract

Knee joint muscle activation imbalances, especially weakness in the vastus medialis oblique, are related to patellofemoral pain within the female population. The available literature presents the leg press as an exercise which potentially targets vastus medialis oblique activation, thus reducing imbalances in the quadriceps muscles. The main aim of the present study was to compare thigh muscle activation and kinematic parameters under different conditions during the inclined leg press exercise in a young female population. A cross-sectional study was conducted on 10 young, trained females. Muscle activation of the vastus medialis oblique, vastus lateralis, rectus femoris and gluteus medialis was analyzed under five different inclined leg press conditions, modifying the feet rotation (0–45° external rotation) and the stance width (100–150% hip width) on the footplate. All the conditions were performed at two different movement velocities: controlled velocity (2″ eccentric–2″ concentric) and maximal intended velocity. Mean propulsive velocity, maximum velocity and maximum power were also assessed. The results show that both controlled velocity conditions and maximal intended velocity conditions elicited a similar muscle activation pattern with greater activation during the concentric phase (*p* < 0.001, ηp^2^ = 0.96). The maximal intended velocity conditions showed greater overall muscle activation (*p* < 0.001, ηp^2^ = 0.91). The vastus medialis oblique presented the greatest muscle activation, followed by the rectus femoris, vastus lateralis and, the gluteus medialis. Furthermore, the inclined leg press condition with 0º feet rotation, 100% hip width distance and the maximal intended velocity generated the greatest kinematic parameter outputs. In conclusion, the inclined leg press exercise might be an optimal exercise to target vastus medialis activation regardless of the feet rotation and stance width conditions.

## 1. Introduction

Vastus medialis oblique (VMO) weakness usually triggers patellar maltracking above the trochlear groove with consequent lateral displacement, increasing the injury risk and its concomitant patellofemoral pain (PFP) [1]. Recent studies have raised concerns about muscle imbalances around the knee joint, their relation to PFP, and their higher rate of occurrence in the female population [1,2]. Accordingly, muscle imbalances amongst quadriceps muscles were exposed as one of the predisposing factors leading to PFP [2]. Therefore, strengthening the VMO might be a strategy for minimizing muscle imbalances around the knee joint, which affect the female population [1,2,3].

In this regard, research on muscle activation using surface electromyography (sEMG) has investigated which exercises preferentially target VMO activation over the vastus lateralis and rectus femoris activation [3,4,5]. Some exercise variants have been tested, from conventional squats and lunges to leg extensions or leg presses [6]. Also, a popular belief has spread that muscle activation might be changed by simply modifying the exercise kinematics, by altering feet rotation or adding adduction/abduction external resistance to conventional exercises [7]. Although most evidence suggests that thigh muscle activation is unlikely to be modified by exercise kinematics, the conclusions are still not compelling [3,5].

The leg press has been presented as an optimal exercise for strengthening the lower limbs, specifically targeting VMO activation, and thus potentially reducing muscle imbalances around the knee joint [8,9]. As yet, few studies have evaluated muscle activation under different conditions during leg press exercises in the female population [10]. Nonetheless, some conditions have been evaluated in the male population. For instance, Escamilla et al. [11] reported no differences in thigh muscle activation under different conditions (stance width and feet rotation modifications) during the leg press. Peng et al. [12], on the other hand, reported higher medial thigh muscle activation in a male population during the leg press with hip adduction resistance, compared to the conventional leg press.

Despite the above, the literature on sEMG assessment of leg press exercise conditions remains scarce, especially in the female population [4,13,14,15]. Muscle activation has only been assessed in the this population while implementing extra hip adduction/abduction resistance conditions during the leg press [4], and different feet height positions on the footplate [14]. However, no study to date has evaluated whether stance width (100–150% hip-width distance) and feet rotation (0–45° external feet rotation) would elicit any changes in muscle activation in the female population when performing the inclined leg press at different movement velocities (controlled velocity and maximal intended velocity).

The kinematic analysis of movement variables as mean propulsive velocity (MPV), maximum velocity (V_max_), and maximum power (P_max_), has been used to gain a deeper insight into performance [16,17,18,19]. For instance, Padulo et al. [20] reported that ballistic performance during leg press largely depended on P_max_ and V_max_ in a male athlete population [21]. Some authors state that velocity could be the best reference for reporting the actual effort during a specific movement [21]. Indeed, preferential use of mean propulsive parameters as MPV, were reported to accurately describe the actual neuromuscular potential of the muscle in a young healthy male population [21]. Thus, MPV, V_max_ and P_max_ have become established as among the most useful kinematic parameters within the performance context [22,23]. However, differences between genders, in respect to kinematic outputs, highlight the necessity to strengthen the evidence in the young female population [13,24,25].

To the best of our knowledge, no study to date has assessed muscle activation (sEMG) along with kinematic parameters during the inclined leg press exercise (and its variants) in a female population. Therefore, the main aims of the present study were: (1) to compare muscle activation of the VMO, vastus lateralis (VL), rectus femoris (RF) and gluteus medialis (GMED) under different conditions of movement velocity (controlled velocity -2″ eccentric and 2″ concentric phase- and maximal intended velocity) and feet placement (0° 100%; 45° 100% and 0° 150%) over the footplate during the inclined leg press exercise; (2) to compare muscle activation under each controlled velocity condition and between contraction phases (eccentric and concentric); and (3) to compare the MPV, V_max_ and P_max_ parameters between the above conditions in a young female population.

## 2. Materials and Methods

### 2.1. Study Design and Participants

A cross-sectional analysis was carried out on a sample population of 10 young, trained women. The mean ±SD for the age, height (cm), body mass (kg), hip width (distance between iliac crests in cm) and one repetition maximum (1RM) for the inclined leg press (kg) of the participants were: 22.6 ± 2.5; 169.0 ± 12.0; 58.3 ± 4.5; 28.0 ± 2.4; 177.5 ± 17.8, respectively. The anthropometric characteristics were measured using a Seca 213 stadiometer (Seca, Hamburg, Germany) for the height, an electronic body composition analyzer (model BF-350; Tanita, Tokyo, Japan) for the weight, and a measuring tape (SECA 200; Harpenden, Holtain Ltd., Wales, UK) for the hip width. The participants had 3.3 ± 2.0 years of resistance training experience (at least 1 year with a minimum of 3 days per week), and had reported no health issues, musculoskeletal injuries or physical limitations over the previous year. They were also familiarized with the inclined leg press exercise. The use of any medications, anabolic steroids or similar drugs was a criterion for study exclusion. The participants voluntarily gave their informed consent to participate in the study. The study protocol was approved by the Research Ethics Committee of the University of Almeria in accordance with the Helsinki Declaration.

### 2.2. Setting

A familiarization and testing session was performed. The participants were prevented from training for a minimum of 48 h before the actual measurements to avoid fatigue bias. All the conditions were performed and the data were recorded within a single session to prevent electrode misplacement.

On the day of testing, the first step was to calculate the resting heart rate (seated for 5′). The next step was to prepare the participants’ skin by shaving, abrading, and cleaning with 96% alcohol and cotton wool. A pre-established warm-up protocol was then performed, consisting of running for 5’ on a treadmill (SALTER RS-30, Salter S.A., Barcelona, Spain) at 60% of the heart rate reserve, calculated by the Karvonen formula ([(maximum heart rate—resting heart rate) * % training sensitive zone] + resting heart rate), and 4 sets of body weight squats and lunges as dynamic movements [26]. The heart rate was tracked using a Polar heart rate monitor (Polar RS400, Polar Vantage NV, Polar Electro Oy, Kempele, Finland).

Subsequently, a progressive approach to the leg press exercise was followed. First, several repetitions were performed with no extra weight. The participants then performed six inclined leg press exercise repetitions with low loads (60 kg) [22]. At this point, electrodes were placed on the right limb over the vastus medialis oblique (VMO), vastus lateralis (VL), rectus femoris (RF) and gluteus medialis (GMED) muscle bellies as required by the Surface Electromyography for the Non-invasive Assessment of Muscles (SENIAM) guidelines [27,28,29,30].

**Figure 1:**
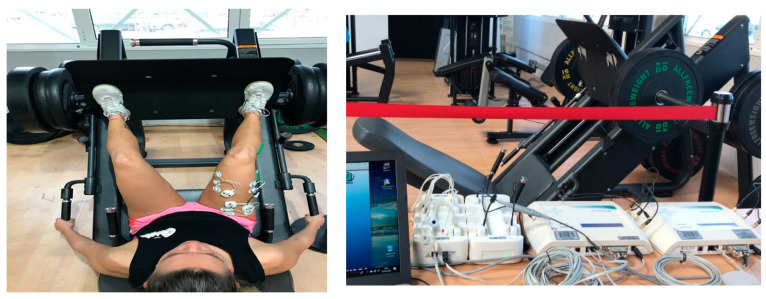
Experimental set-up.

As the electrodes and electrogoniometer were being placed, the strength and conditioning professional briefly reminded the participants of the technique for performing the conditions. The initial position was set at a 0° knee flexion (extended knees) and the participants were asked to bend their knees in a controlled manner up to a 90° knee flexion position (shinbone parallel to the floor) and 60° hip joint position, which was set as the turnover point. Tape adhesive was stuck to the inclined leg press machine at this turnover point to provide a visual orientation for the participant. The feet were positioned at medium height on the leg press footplate (Figure 1). Immediately after a brief reminder on the technique, we proceeded to test the 1RM leg press individual load.

### 2.3. Approach to 1RM

A load progression test was performed to find out the 1RM of each participant. Up to eight sets were performed at a maximal intended velocity until the 1RM load was reached [22]. The initial load was 60 kg. Four repetitions were performed with a 3–4′ rest for those sets with a velocity up to 1.15 m·s^−1^ MVP. For medium loads (1.15 m·s^−1^ ≥ MPV ≥ 0.5 m·s^−1^), two repetitions were performed with a 5′ rest; and for maximum loads (MPV < 0.5 m·s^−1^), only one repetition was performed with a 6′ rest [22].

As reported elsewhere [22], an increment of 10% of 1RM was added while going through the incremental sets, until the participant achieved a MVP of 0.5 m·s^−1^ [21,22]. After that, the increments ranged from 1.25 kg to 5 kg (AZAFIT BUMPER PLATES, Viseu, Portugal) until the participant reached the 1RM load. After performing this approach to the 1RM procedure, the 1RM load was set as the one that each participant could lift only once [22,23].

Real-time velocity feedback was provided to the participants (T-Force System, Ergotech, Murcia, Spain) and examiners delivered verbal encouragement to ascertain the maximal intended velocity [26,31]. Immediately after the 1RM test, the participants proceeded to perform the five different leg press conditions while the examiners recorded the data. During the maximal intended velocity sets, a self-selected controlled velocity was established for the eccentric phase, with a 1–2 s pause between phases to avoid elastic energy accumulation [22,23]; whereas for the concentric phase, maximum effort (“as fast as possible movements”) were encouraged.

### 2.4. Outcome Variables

The main outcome variable was muscle activation, collected through sEMG. The MPV, Vmax and Pmax kinematic parameters were also assessed. Furthermore, an electrogoniometer was used to accurately identify the different repetitions.

The five leg press variants performed were as follows:

At a controlled velocity of 2″ eccentric phase and 2″ concentric phase:
  Leg press at 0° forefoot external rotation and 100% hip width distance stance (0° 100%);
  Leg press at 45° forefoot external rotation and 100% hip width distance stance (45° 100%);
  Leg press at 0° forefoot external rotation and 150% hip width distance stance (0° 150%).
At maximal intended velocity:
  Leg press at 0° forefoot external rotation and 100% hip width distance stance;
  Leg press at 45° forefoot external rotation and 100% hip width distance stance.

The hip width was taken as the distance between the iliac crests (in cm). All the conditions were performed at a relative intensity of 70% 1RM [10,32,33]. For each condition set, six repetitions were recorded, leaving a 4′ rest between sets. All the variables were recorded throughout the process, including the approach to 1RM.

### 2.5. Materials

An inclined leg press device was used (FITTECH PL688, Viseu, Portugal) located in the University of Almeria’s Sports Center. For the sEMG, bipolar adhesive Ag/AgCl electrodes (Medico Lead-Lok, Noida, India) were used, leaving 2 cm between each positive and negative pair, in accordance with the SENIAM guidelines.

The raw sEMG signal from the targeted muscles was recorded with a WBA Mega device (Mega Electronics Ltd., Kuopio, Finland) at a sampling frequency of 1000 Hz. The analog signal was converted to digital via an A/D converter (National Instruments, New South Wales, Australia) and filtered by bandwidth (12–450 Hz) with a fourth-order Butterworth filter using the LabView software program (National Instruments, Austin, TX, USA). The raw sEMG signals were then converted into root-mean-square (RMS) signals with 20-millisecond windows for further analysis using the MEGAWIN software program (Mega Electronics Ltd.). The maximum voluntary contraction (MVC) of each muscle was recorded during the 1RM test. Maximum peak muscle activation in microvolts (µV) at intervals of one second was calculated during the MVC for each muscle; this was used to normalize the sEMG [34,35].

The kinematic parameters were extracted from the recorded data by linear transducer sampling at 1000 Hz (T-Force System, Ergotech). The electrogoniometer (Biometrics Ltd., Newport, UK) was also connected and synchronized to the WBA Mega device, allowing each repetition to be identified. The wave described by the goniometer ranged from 0° (knees fully extended) to 90° (maximum knee flexion allowed). Each interval between 0–90° described the eccentric phase, and each interval between 90–0° described the concentric phase for a single repetition.

Finally, a KORG MA-1 (Keio Electronic Laboratories, Tokyo, Japan) metronome was used for the controlled velocity sets. A pre-established velocity of 60 bpm (2″ for each phase with no rest between them) was set to ensure the controlled velocity.

### 2.6. Statistical Analyses

Normality was assessed through the Shapiro–Wilk test. As all the variables presented a normal distribution, the parametric tests were then performed. Descriptive statistics were used to extract the mean values and standard deviations of the dependent variables. The one-way random effects model was used to obtain the relative reliability of the measurements using the ICC with a 95% confidence interval.

The sample size and statistical power were calculated with the G*power 3.1 for Mac OS X software program [36]. The statistical power was >0.9 for all the variables analyzed with the sample size used in the current study. For the paired samples t-test, the effect size was calculated with Cohen’s d [37] using the G*power 3.1 for Mac OS X software program [36]. An effect size of d < 0.2 was considered small, d at approximately 0.5 was considered moderate, and d > 0.8 was considered large.

Additionally, Mauchly’s sphericity test was performed after the ANOVA analyses. If an assumption was violated, a Greenhouse-Geisser correction was performed. The Bonferroni post hoc adjustment was employed when a significant main effect was observed within pairwise comparisons. In addition, the effect sizes (ES) were calculated for each ANOVA using the partial eta-squared (ηp²) ratio of variance; settings of 0.2, 0.5, 0.8, and 1.3 were established as lower thresholds for “small”, “medium”, “large”, and “very large” effect sizes, respectively. IBM SPSS software (v.26) was used to run the statistical analyses with the level of significance set at alpha 0.05.

A 2 * 2 * 4 * 2 (exercise*contraction type*muscle*velocity) repeated measures ANOVA was performed to determine the differences in muscle activation, as a percentage of the maximum voluntary contraction (%MVC) between the conditions, according to the exercise velocity and the feet placement on the footplate. Moreover, a 3 * 2 * 4 (exercise*contraction type*muscle) repeated measures ANOVA was applied to determine the differences in muscle activation (%MVC) within each controlled velocity condition during both the eccentric and concentric phases.

A paired samples *t*-test was performed to determine the differences in MPV and Vmax between the two conditions executed at maximal intended velocity (0° 100% and 45° 100%). Moreover, two separate repeated measures ANOVA were employed to determine: (1) the differences in the Pmax between the three conditions executed at the controlled velocity (0° 100%, 45° 100% and 0° 150%); and (2) the differences in the Pmax between all the conditions according to the velocities (velocity*exercise).

## 3. Results

### 3.1. sEMG

**Figure 2:**
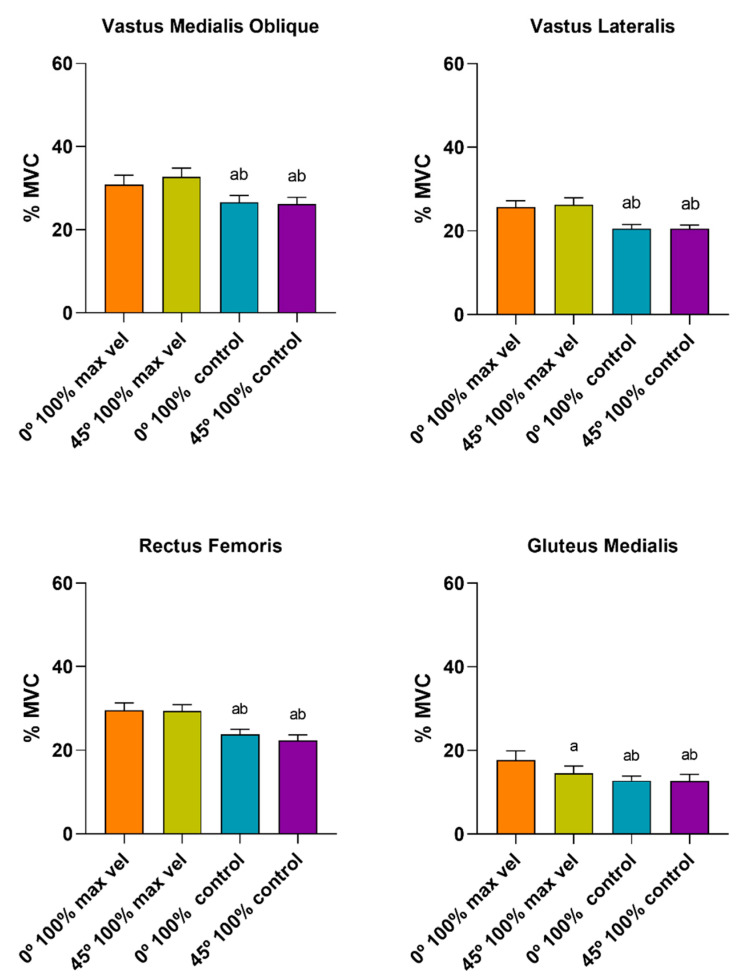
Comparison of eccentric muscle activation for each muscle between 0° 100% and 45° 100%, performed at the maximal intended and controlled velocities (^a^ difference from 0° 100% max. vel., ^b^ difference from 45° 100% max. vel.; *p* < 0.05).

**Figure 3:**
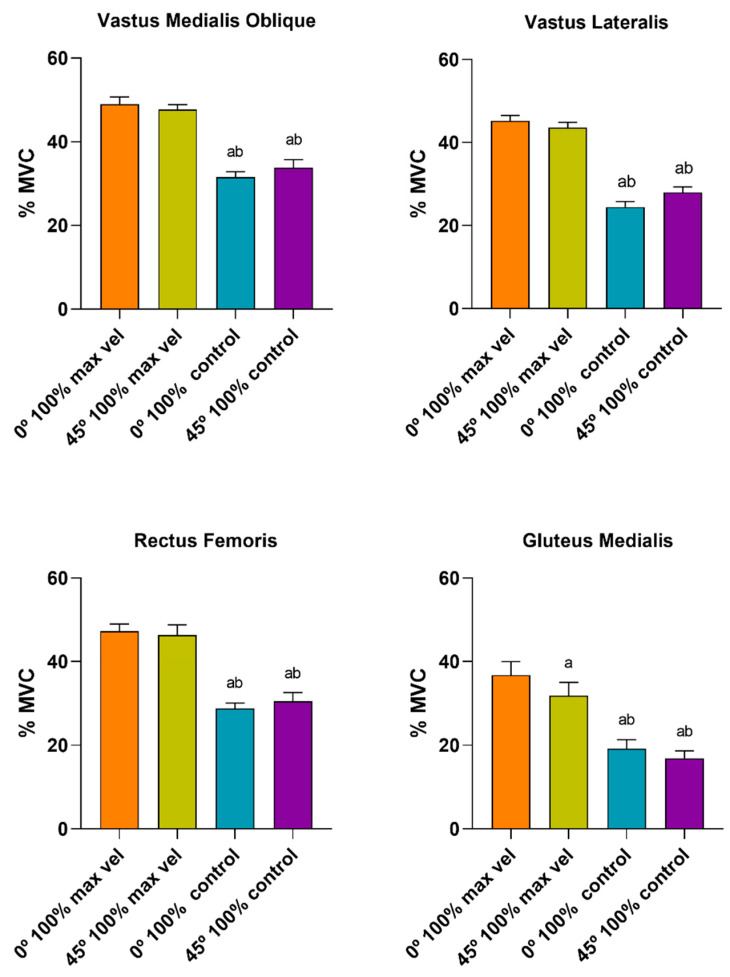
Comparison of concentric muscle activation for each muscle between 0° 100% and 45° 100%, performed at the maximal intended and controlled velocities (^a^ difference from 0° 100% max. vel., ^b^ difference from 45° 100% max. vel.; *p* < 0.05).

Figure 2 and Figure 3 compare the muscle activation of the maximal and controlled velocities under the 0° 100% and 45° 100% conditions. The ANOVA showed a significant main effect/interaction for the contraction type (F_(1, 9)_ = 215.66, *p* < 0.001, ηp^2^ = 0.96), the muscle (F_(3, 27)_ = 95.81, *p* < 0.001, ηp^2^ = 0.91), the velocity (F_(1, 9)_ = 97.04, *p* < 0.001, ηp^2^ = 0.91), the contraction type*muscle (F_(3, 27)_ = 21.52, *p* < 0.001, ηp^2^ = 0.70), the exercise*velocity (F_(1, 9)_ = 5.43, *p* < 0.045, ηp^2^ = 0.37), the contraction type*velocity (F_(1, 9)_ = 78.50, *p* < 0.001, ηp^2^ = 0.89), the exercise*contraction type*velocity (F_(1, 9)_ = 26.43, *p* = 0.001, ηp^2^ = 0.74), the muscle*velocity (F_(3, 27)_ = 25.71, *p* < 0.001, ηp^2^ = 0.74), the contraction type*muscle*velocity (F_(3, 27)_ = 10.87, *p* < 0.001, ηp^2^ = 0.54), and the exercise*contraction type*muscle*velocity (F_(3, 27)_ = 4.22, *p* < 0.014, ηp^2^ = 0.31).

When comparing the muscle activation pattern between the conditions (0° 100% and 45° 100% at the maximal intended and controlled velocities), significant differences were only shown for the GMED—between 0° 100% and 45° 100% under the maximal intended velocity conditions for both the eccentric and concentric phases (Figure 2 and Figure 3). The VMO, VL and RF presented similar muscle activation patterns under the different conditions. Therefore, one can observe that the 45° external feet rotation did not elicit significant changes in muscle activation.

Overall, greater muscle activation was elicited under maximal intended velocity conditions. Under all the conditions, muscle activation was greater in the concentric phase than in the eccentric phase for all muscles evaluated, except for the VMO at 0° 100% under controlled velocity conditions.

**Figure 4:**
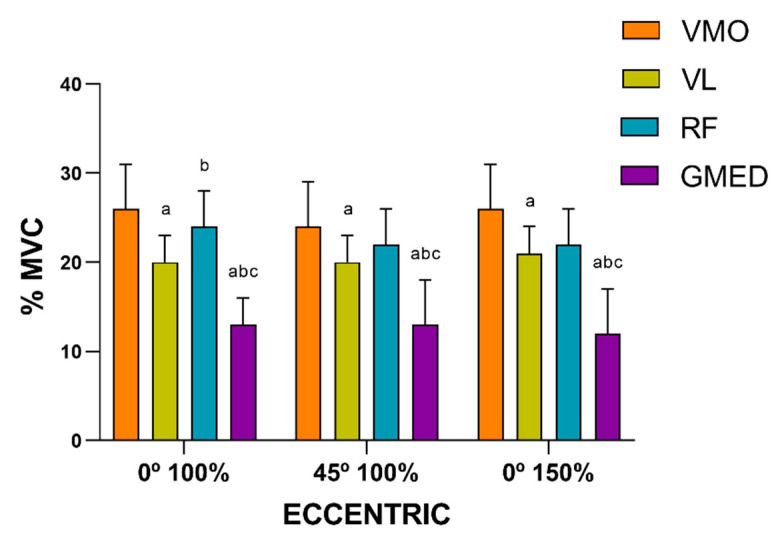
Comparison of the muscle activation (expressed in %MVC) between the muscles during the eccentric phase under each condition, performed at the controlled velocity (^a^ difference from VM, ^b^ difference from VL, ^c^ difference from RF; *p* < 0.05). VMO: vastus medialis oblique; VL: vastus lateralis; RF: rectus femoris; GMED: gluteus medialis.

**Figure 5:**
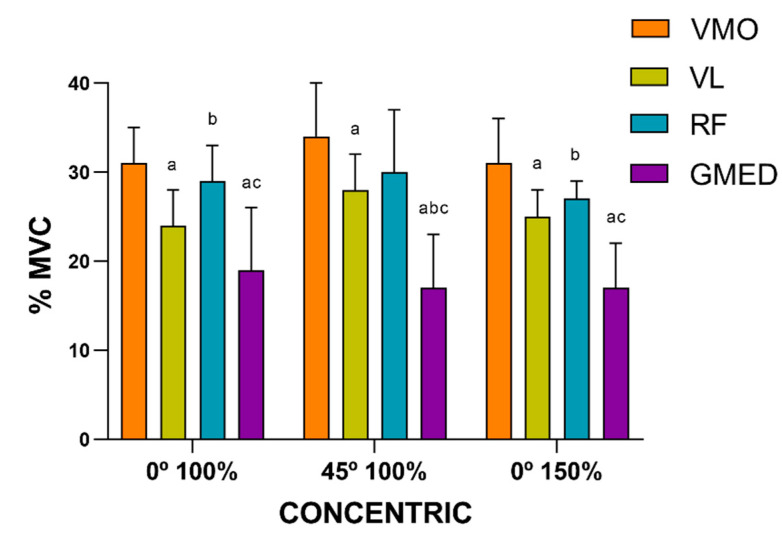
Comparison of the muscle activation (expressed in %MVC) between the muscles during the concentric phase under each condition, performed at the controlled velocity (^a^ difference from VM, ^b^ difference from VL, ^c^ difference from RF; *p* < 0.05). VMO: vastus medialis oblique; VL: vastus lateralis; RF: rectus femoris; GMED: gluteus medialis.

**Figure 6:**
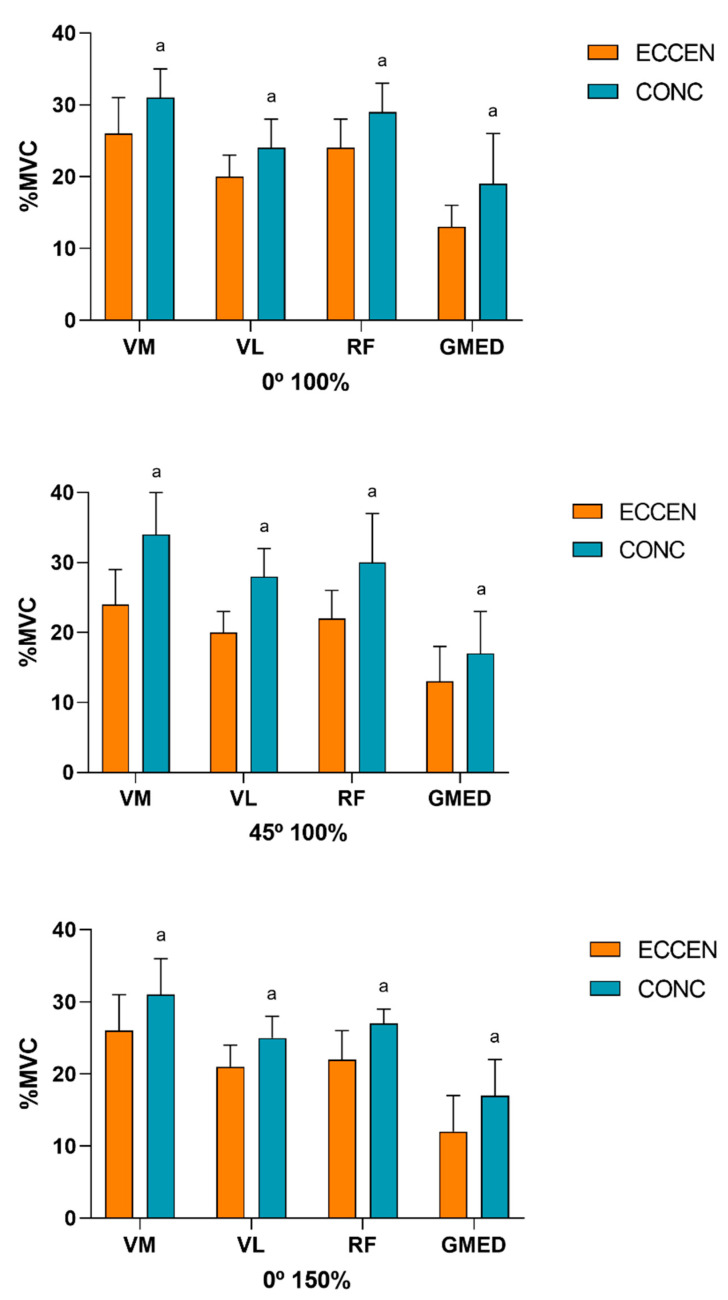
Comparison of the muscle activation (expressed in %MVC) between phases for each muscle under each controlled velocity condition (^a^ difference from the eccentric phase; *p* < 0.05). VMO: vastus medialis oblique; VL: vastus lateralis; RF: rectus femoris; GMED: gluteus medialis. ECCEN: eccentric; CONC: concentric.

Figure 4, Figure 5 and Figure 6 compare the muscle activation between the muscles during the eccentric and concentric phases under each condition, performed at the controlled velocity. The ANOVA showed significant main effects for the contraction type (F_(1, 9)_ = 33.26, *p* < 0.001, ηp^2^ = 0.78) and the muscle (F_(3, 27)_ = 31.77, *p* < 0.014, ηp^2^ = 0.77).

No differences were found between the distinct muscles under the conditions tested (0° 100%, 45° 100% and 0° 150%). In terms of %MVC, a similar muscle activation pattern was presented for both the eccentric and concentric phases under the three conditions tested. However, the VMO showed the greatest muscle activation, followed by the RF and VL, whilst the GMED muscle presented the least muscle activation (Figure 4 and Figure 5). Under the three conditions tested, at the controlled velocity, muscle activation was greater during the concentric phase than during the eccentric phase (Figure 6).

### 3.2. Kinematic Parameters

The 1RM load was reached at a mean velocity of 0.22 ± 0.02 m·s^−^¹ for all the participants. Table 1 compares the MPV and V_max_ between the conditions executed at maximal velocity. The paired samples *t*-test showed a higher MPV and V_max_ under the 0° 100% condition than under the 45° 100% condition.

The repeated measures ANOVA showed no main effect on P_max_ under the three conditions executed at the controlled velocity (F_(2, 18)_ = 1.91, *p* = 0.194, ηp^2^ = 0.27). Thus, the P_max_ exhibited no significant differences between the three conditions (Table 2).

Table 3 compares the P_max_ between the conditions executed at both the maximal and controlled velocities. The repeated measures ANOVA showed a main effect/interaction on P_max_ for the velocity (F_(1, 9)_ = 153.40, *p* < 0.001, ηp^2^ = 0.94) and the velocity*exercise (F_(1, 9)_ = 12.13, *p* = 0.007, ηp^2^ = 0.57).

## 4. Discussion

The main objectives of the current study were to compare the activation of distinct muscles under different conditions, in terms of movement velocity and feet placement on the footplate, during the inclined leg press exercise, and to compare muscle activation within each controlled velocity condition and between the contraction phases. Regarding muscle activation, the main outcomes revealed a similar muscle activation pattern for the 0° 100%, 45° 100% and 0° 150% conditions, with no preferential activation of a single muscle based on these conditions. The greatest overall muscle activation was shown under maximal intended velocity conditions. Furthermore, muscle activation was greater for all the concentric phases than for the eccentric phases, regardless of the inclined leg press conditions; this outcome is broadly supported by the literature [4,27,38]. The VMO elicited the greatest muscle activation in terms of %MVC, followed by the RF, VL and GMED muscles. The similar results for the muscle activation pattern, with no preferential muscle activation reported for the different muscles under the conditions tested, might skew the purported importance of the feet stance [7].

A previous study conducted on a female population agreed with our results regarding muscle activation—Da Silva et al. [14] reported similar muscle activation for the RF and VL during the flat leg press and the inclined leg press performed at an 80% 1RM intensity in a young female population. They also encouraged the most comfortable feet stance on the footplate, but they did not analyze VMO muscle activation, thus providing an incomplete picture of overall quadriceps muscle activation during those exercises. In contrast, Machado et al. [4] reported conflicting results. They compared VMO, VL and RF muscle activation during the inclined leg press (with two modifications) in a young female population. They found greater VMO muscle activation during the leg press when it was performed with a physio ball between the knees (extra hip adduction) than when the leg press was performed with an elastic band around the knees. However, their results should be interpreted with caution, since the exercises were performed at a low intensity (70% of 10RM) and the resistance training experience of the participants was not reported. Familiarization and exercise intensity could substantially modify the muscle activation elicited [29,39,40]. Our results only agreed in that the greatest muscle activation was elicited during the concentric phase.

The present study results are consistent with other studies in the male population. For example, Escamilla et al. [11] affirmed that varying the foot external rotation (0–30°) during the flat leg press did not affect VMO, VL or RF muscle activation in a young male population. Peng et al. [12], on the other hand, analyzed VM, VL and hip adductor longus muscle activation during the leg press with isometric hip adduction resistance in a young male population. They reported greater adductor longus muscle activation than when performing the conventional leg press. Nevertheless, preferential VMO activation with this modification was not supported. In addition, in their systematic review, Smith et al. [5] stated that the VMO muscle could not be preferentially activated by modifying the lower limb position during quadriceps exercises. However, they did not analyze leg press exercises in their review, thus highlighting the need to expand our knowledge on this issue [8,11].

Regarding kinematics, the purpose of the present study was to compare MPV, V_max_ and P_max_ kinematic parameters under the different conditions. The study results showed higher MPV, V_max_ and P_max_ during the 0° 100% exercise than during the 45° 100% exercise when performed at the maximal intended velocity. There is only one previous study that analyzed knee kinematics during the leg press exercise in a young male population [11]. They concluded that varying the feet rotation (0°–30°) and feet stance (narrow and wide) did not affect the knee forces during the flat leg press exercise, so participants were encouraged to adopt their preferred feet stance. Although we did not analyze knee forces, they might relate to our results to some degree, given that we reported higher kinematic parameters for the 0° 100% maximal intended velocity condition; this was recognized by all the participants as the most comfortable position. Since muscle activation showed no significant differences depending on feet stance or feet external rotation, and that the kinematic parameters were better for the 0° 100% maximal intended velocity condition, we would recommend that participants adopt their preferred stance during the inclined leg press exercise for maximizing performance categories. In contrast, the Pmax presented no significant differences between the 0° 100%, 45° 100% and 0° 150% conditions performed at controlled velocity. This is clearly explained by the controlled velocity we imposed during the controlled velocity sets, which prevented the participants from applying greater power to the movement.

One of the main limitations of the present study is the small sample population. Although the statistical power was excellent for all the variables analyzed with the sample size used, we only assessed 10 young women, so the findings should be interpreted with caution. Regarding the different conditions assessed, it was possible to perform a 0° and 45° external feet rotation with a 100% hip width stance at the controlled and maximal intended velocities. However, the 45° external feet rotation and maximal intended velocity conditions were impossible to perform with a 150% hip width stance since the inclined leg press dimensions (the footplate size) were not favorable.

## 5. Conclusions

To sum up the main findings:

The muscle activation pattern showed no differences between conditions (0° 100%, 45° 100% and 0° 150% at both the controlled and maximal intended velocities). However, the inclined leg press conditions performed at maximal intended velocity did elicit greater overall muscle activation than those conditions performed at the controlled velocity.

Muscle activation was greater during the concentric phases than the eccentric phases under all the conditions. All the conditions presented a similar muscle activation pattern.

The VMO showed greater muscle activation in terms of %MVC compared to the VL, RF and GMED. Therefore, the inclined leg press exercise could be an exercise of choice when the target is to elicit VMO activation to reduce knee muscle imbalances.

The MPV, V_max_ and P_max_ were greater during the 0° 100% maximal intended velocity sets, which the participants reported as their preferred condition. This fact, together with the similar muscle activation pattern between the conditions, leads us to conclude that the most comfortable, self-selected stance should be encouraged.

## Figures and Tables

**Table 1:** Mean, SD, *p*-value and effect size for the MPV and V_max_ comparing the conditions executed at the maximal velocity.

	Mean ± SD		*p*-Value	Effect Size (*d*)
	Maximal Velocity 0° 100%	Maximal Velocity 45° 100%		
MPV (m·s^−^¹)	0.37 ± 0.06	0.33 ± 0.06	0.001	0.66
V_max_ (m·s^−^¹)	0.75 ± 0.09	0.71 ± 0.09	<0.001	0.44

**Table 2:** Mean, SD, *p*-value and effect size for the P_max_ comparing the conditions executed at the controlled velocity.

	Mean ± SD			*p*-Value
	0° 100%	45° 100%	0° 150%	
P_max_ (W)	286.6 ± 47.8	322.6 ± 91.1	291.8 ± 65.9	>0.050

**Table 3:** Mean, SD, *p*-value and effect size for the P_max_ comparing the conditions executed at both the maximal and controlled velocities.

		Mean ± SD		*p*-Value	Effect Size (*d*)
		0° 100%	45° 100%		
P_max_ (W)	Maximal velocity	1037.4 ± 211.0	973.2 ± 213.8	<0.001	0.47
	Controlled velocity	286.6 ± 47.8	322.6 ± 91.1	0.179	0.45
